# Investigation of *ospC* Expression Variation among *Borrelia burgdorferi* Strains

**DOI:** 10.3389/fcimb.2017.00131

**Published:** 2017-04-20

**Authors:** Xuwu Xiang, Youyun Yang, Jimei Du, Tianyu Lin, Tong Chen, X. Frank Yang, Yongliang Lou

**Affiliations:** ^1^Key Laboratory of Laboratory Medicine, Ministry of Education of China, School of Laboratory Medicine, Wenzhou Medical UniversityWenzhou, China; ^2^Department of Microbiology and Immunology, Indiana University School of MedicineIndianapolis, IN, USA; ^3^College of Arts and Sciences, University of PennsylvaniaPhiladelphia, PA, USA

**Keywords:** *Borrelia burgdorferi*, OspC, endogenous plasmid, multiplex PCR, BosR

## Abstract

Outer surface protein C (OspC) is the most studied major virulence factor of *Borrelia burgdorferi*, the causative agent of Lyme disease. The level of OspC varies dramatically among *B. burgdorferi* strains when cultured *in vitro*, but little is known about what causes such variation. It has been proposed that the difference in endogenous plasmid contents among strains contribute to variation in OspC phenotype, as *B. burgdorferi* contains more than 21 endogenous linear (lp) and circular plasmids (cp), and some of which are prone to be lost. In this study, we analyzed several clones isolated from *B. burgdorferi* strain 297, one of the most commonly used strains for studying *ospC* expression. By taking advantage of recently published plasmid sequence of strain 297, we developed a multiplex PCR method specifically for rapid plasmid profiling of *B. burgdorferi* strain 297. We found that some commonly used 297 clones that were thought having a complete plasmid profile, actually lacked some endogenous plasmids. Importantly, the result showed that the difference in plasmid profiles did not contribute to the *ospC* expression variation among the clones. Furthermore, we found that *B. burgdorferi* clones expressed different levels of BosR, which in turn led to different levels of RpoS and subsequently, resulted in OspC level variation among *B. burgdorferi* strains.

## Introduction

Lyme disease is the most common arthropod-born disease in the United States, Europe as well as Asia (Samuels, 2011; Radolf et al., 2012). The causative agent, *Borrelia burgdorferi*, is a spirochete that has a dual-membrane cell envelope. Unlike other Gram-negative bacteria that have lipopolysaccharide (LPS) on the outer membrane, *B. burgdorferi* outer membrane lacks LPS and contains numerous lipoproteins. These outer surface lipoproteins are differentially expressed and play key roles in host adaptation during its enzootic cycle between ticks and mammals (Radolf et al., 2012; Caimano et al., 2016). For example, the major outer surface lipoprotein C (OspC), is induced in *B. burgdorferi* during nymphal tick feeding and during early phase of mammalian infection, and it plays an essential role for spirochetes to establish early infection and may also be important for spirochetal transmission from tick to mammal (Grimm et al., 2004b; Pal et al., 2004; Carrasco et al., 2015a).

The genome of *B. burgdorferi* comprises of more than 21 linear and circular endogenous plasmids with 5–56 kb in size (Fraser et al., 1997; Casjens et al., 2000, 2012), making its genome have the largest plasmid content among any reported bacteria (Chaconas and Norris, 2013). Many endogenous plasmids encode outer surface lipoproteins, and propagation of spirochetes *in vitro* can lead to plasmid loss, which often results in attenuation of infection in mice or a defect in ticks (Purser and Norris, 2000). As such, a crucial step for genetic manipulation of *B. burgdorferi* is to perform plasmid profile for each clone to be sure the phenotype is not due to a plasmid loss. In addition, studies on the correlation between the loss of a particular plasmid and an *in vitro* or *in vivo* phenotype have led to identification of several virulence-associated genes (Purser et al., 2003; Revel et al., 2005). However, the contribution of each plasmid-encoded genes to the life cycle of *B. burgdorferi* remains largely unknown.

*B. burgdorferi* sensu lato (s.l.) complex includes *B. burgdorferi* sensu stricto (s.s.), *B. afzelii*, and *B. garinii*. *B. afzelii*, and *B. garinii* are found outside of the United States. *B. burgdorferi* s.s. are highly heterogeneous with significant variation in virulence and endogenous plasmid content. *B. burgdorferi* s.s. strain B31, which was isolated from a tick collected on Shelter Island (Burgdorfer et al., 1982), is the first sequenced strain and has been widely used for Lyme disease research. Another widely used *B. burgdorferi* strain is strain 297, which was isolated from the cerebral spinal fluid (CSF) sample of a Lyme borreliosis patient (Steere et al., 1983; Hughes et al., 1992). The endogenous plasmid sequence of *B. burgdorferi* strain 297 was published in 2011(Schutzer et al., 2011).

The advantage of using *B. burgdorferi* strain 297 to study gene regulation of *B. burgdorferi* is that *ospC* is highly expressed under the *in vitro* cultivation condition. Expression of *ospC* is induced by elevated temperature (37°C relative to ambient temperature, 25°C), low pH (pH 6.8–7.0), and high cell density (Yang et al., 2000). In fact, while high level of *ospC* expression can be achieved in *B. burgdorferi* strain B31 by temperature shift (first at 25°C then shift to 35°C), *ospC* expression in strain 297 can be easily achieved by directly cultivating the spirochete at 35 or 37°C without temperature shift. The level of OspC in 297 strain can be readily visualized as one of the most dominant bands in the protein profile when separated on Coomassie stained gel. This advantage has contributed in part, to the discovery of the first regulatory pathway in *B. burgdorferi*, the Rrp2-RpoN-RpoS pathway. In this pathway, the two-component system Hk2-Rrp2 activates the σ^N^–σ^S^ (RpoN-RpoS) sigma factor cascade, leading to the production of σ^S^ (RpoS), which in turn, controls expression of many virulence genes including *ospC* and many other genes (for review, see, Samuels, 2011; Radolf et al., 2012; Ouyang et al., 2014; Ye et al., 2016b). In addition, many other factors including BosR (Hyde et al., 2009; Ouyang et al., 2009, 2011), BadR (Miller et al., 2013; Ouyang and Zhou, 2015), DsrA (Lybecker and Samuels, 2007), Rrp1 (Rogers et al., 2009; Sze et al., 2013; He et al., 2014), BtmA (Troxell et al., 2013), SodA (Esteve-Gassent et al., 2015), BBD18 (Dulebohn et al., 2014), also influence *rpoS* and *ospC* expressions.

Performing plasmid profile for strain 297 has been challenging, as the endogenous plasmid sequences for this strain were not available until recently. In the past, primers used for tracking most of the plasmids of strain 297 clones were designed based on the sequence of strain B31, which is not appropriate. As such, it is difficult to determine whether the parental low passage 297 strain is a single or mixed clones. Similarly, it is also difficult to determine whether the commonly used 297 clones derived from the original 297 strain have a complete plasmid content. In this study, based on published plasmid sequences of *B. burgdorferi* strain 297 (Schutzer et al., 2011; Casjens et al., 2012), we developed a multiplex PCR method specifically for rapid plasmid profiling of strain 297. We analyzed plasmid profiles of the original 297 strain and the derived clones with different levels of *ospC* expression. Since it has been reported that genes carried on certain plasmids in strain B31, e.g., *bbd18* on lp17, influence *ospC* expression (Sarkar et al., 2011; Dulebohn et al., 2014), we tested whether the difference in plasmid contents contributes to the variation in OspC levels among the clones. We found that different clones expressed different levels of BosR, and such difference, not the difference in their plasmid profiles, contributes to the *ospC* expression variation among *B. burgdorferi* strains.

## Materials and methods

### *B. burgdorferi* strains and culture conditions

The parental *B. burgdorferi* strain 297 is a non-clonal strain, originally obtained from culturing the CSF sample of a Lyme borreliosis patient (Steere et al., 1983; Hughes et al., 1992). No more than three passages from the original strain was used in this study. AH130 and PL133 were derived clones from original strain 297. Spirochetes were cultivated in complete Barbour-Stoenner-Kelly-II (BSK-II) medium (Barbour, 1984) at 37°C with 5% CO_2_. Semi-solid agar plating of *B. burgdorferi* was carried out as previously described [complete Barbour-Stoenner-Kelly- II (BSK-II) medium with 2.5% agar] (Samuels, 1995). Types of colony morphology were observed 8–12 days after plating.

### Sodium dodecyl sulphate-polyacrylamide gel electrophoresis (SDS-PAGE) and immunoblotting

The method for SDS-PAGE was described previously (Carrasco et al., 2015b). Briefly, spirochetes were cultured from 10^4^ cells/ml and harvested at day 7 (stationary phase 10^8^ cells/ml) by centrifugation at 8,000 g for 10 min and washed two times with PBS (pH 7.4) at 4°C. Pellets were suspended in SDS buffer containing 50 mM Tris-HCl (pH 8.0), 2% sodium dodecyl sulfate (SDS), and 10 mM dithiothreitol (DTT). Cell lysates (5 × 10^7^ cells per lane) were separated by 12% SDS-PAGE and stained with Coomassie blue or transferred to nitrocellulose membranes (GE-Healthcare, Milwaukee, WI). Membranes were blotted with monoclonal antibodies against FlaB, RpoS, and BosR (He et al., 2008; Xu et al., 2010; Troxell et al., 2013) with 1:1,000, 1:50, and 1:500 dilutions, respectively, and then with goat anti-mouse lgG-HRP secondary antibody (1:1,000, Santa Cruz Biotechnology). Detection of horseradish peroxidase activity was determined using enhanced chemiluminescence method (Thermo pierce ECL Western Blotting Substrate), and subsequently by exposure to X-ray film.

### Primer design

Multi-alignment among all 19 endogenous plasmid sequences of *B. burgdorferi* strain 297 (Table 1) were performed using ALIGNMENT tool in Software Vector NTI (Thermo Fisher Scientific, CA). The identified unique sequences of each primer pair for PCR of each plasmid were then subjected to BLAST analysis (National Center for Biotechnology Information) against all published *Borrelia* chromosomal sequences in order to confirm no potential homology with chromosomal sequence (note that *B. burgdorferi* strain 297 chromosomal sequence is not available). General guidelines for primer design were as follows: (a) Primers should be at least 20 nucleotides but not longer than 28 nucleotides, to minimize the possibility of primer-dimer formation and unspecific binding; (b) CG nucleotides should be distributed equally on both ends; (c) All PCR products should differ in size by at least 20 bp in order to allow efficient separation by gel electrophoresis. See primer sequences in Tables 2, 3.

**Table 1:** **List of endogenous plasmids in *B. burgdorferi* strain 297**.

**Linear or circular plasmids**	**Designation**	**Gene bank**	**Size(bp)**	**CG(%)**
lp17	D	GI: 410108990	12,965	24.1
lp25^a^	E	/	/	/
lp28-1	F	GI: 410108985	20,978	30.7
lp28-3	H	GI: 410108988	25,211	25.0
lp28-4	I	GI: 410108991	25,788	24.5
lp28-5	Y	GI: 410108986	20,974	24.3
lp28-6	Z	GI: 410108984	22,135	33.0
lp36	K	GI: 410108983	22,715	25.7
lp38	J	GI: 410108989	27,035	25.4
lp54	A	GI: 256041893	48,220	28.3
cp26	B	GI: 410108980	26,514	26.2
cp32-1	P	GI: 410108993	30,902	29.2
cp32-3	S	GI: 410108995	30,262	28.8
cp32-4	R	GI: 410108981	30,301	29.2
cp32-5	V	GI: 410108979	30,636	29.2
cp32-6	M	GI: 410108976	30,641	29.1
cp32-7^b^	O	GI: 410108978	21,165	28.8
cp32-9^b^	N	GI: 410108975	21,172	28.3
cp32-11	W	GI: 410108994	30,286	28.9
cp32-12	X	GI: 410108974	30,795	29.3

a *The sequence of lp25 of 297 strain is not available*.

b *cp32-7 and cp32-9 are previously named as cp18-1 and cp18-2, respectively*.

**Table 2:** **PCR primer sequences for each linear plasmid**.

**Linear plasmid**	**Sequence 5′->3′**	**Length (bp)**	**Coordination 5′–3′**	**Tm (°C)**	**Concentration in 12.5 × primer mix (uM)**	**Amplicon size (bp)**
lp25F^*^	CGTTATCTACCGTTTATAGGTTTGA	25	266–290	52.5	2.7	100
lp25R^*^	TTGAAACCTTAGCATCTTCAAATCCTT	27	367–341	55.3	2.7	
lp54F	TCTTAATATCAACCTAGAATATTCC	25	965–989	48.0	2.7	125
lp54R	TAACAGACGAAGAAGAAGAGACTTT	25	1,089–1,065	53.7	2.7	
lp28-6F	ATTGATGAATGGCGTTACCATTAGT	25	11,486–11,510	54.9	2.25	151
lp28-6R	CATTGTATCAGACATAACACTTCAT	25	11,636–11,612	51.2	2.25	
lp28-4F	CTTGCGATCTACCAACAATGAGTAA	25	27–51	55.1	2.7	175
lp28-4R	AGTTTATCTGATATTAGGAGATAGT	25	201–177	48.5	2.7	
lp28-1F	TCAATCAAACATATTGGGTGAAGAA	25	53,00–5,324	53.0	2.25	200
lp28-1R	TTGCCTGTATTGCTAAATTACTATG	25	5,499–5,475	51.5	2.25	
lp28-3F	TGTCTAAGGAAGGTTTAAGGCTTAT	25	21,131–21,155	53.8	2.25	225
lp28-3R	AGAAATGCAGTGCTTGCGTCTAAAT	25	21,355–21,331	57.6	2.25	
lp28-5F	TTAGGAACTCTGACTATCATGGAAT	25	5,175–5,199	53.2	2.25	251
lp28-5R	AAGGCGTCATTACAATTATCTAAACA	26	5,425–5,400	53.0	2.25	
lp17F	AGCGAAGAATTATTCTTGCAATGTG	25	12,507–12,531	54.4	2.25	275
lp17R	CGACTTCTTATATAGCTGAGATTCT	25	12,781–12,757	51.7	2.25	
lp38F	AATCCAGGTATTCTTGTTGCTGGTC	25	20,896–20,920	57.2	3.38	325
lp38R	TTATTAGGAGACGATATTAATATAA	25	21,220–21,196	45.0	3.38	
lp36F	AAGTGGTGAATTGGAGGAGCCTATT	25	15,394–15,418	58.2	2.25	369
lp36R	TTAGCAAAGTTGTCAAGGCGTGTAGA	26	15,762–15,737	58.5	2.25	

* *The primer sequences for lp25 were based on bptA gene sequence*.

**Table 3:** **PCR primer sequences for each circular plasmid**.

**Circular plasmid**	**Sequence 5′->3′**	**Length(bp)**	**Coordination 5′–3′**	**Tm (°C)**	**Concentration in 12.5 × primer mix (uM)**	**Amplicon size (bp)**
cp32-4F	TGAGCAGCACAAGTAGATGATGCTT	25	27,800–27,824	58.6	2.38	121
cp32-4R	CCGGGGATAATGCTAGTCAACAA	23	27,920–27,898	56.8	2.38	
cp32-5F	AAGGTGCTTTAGACACAAGAGATGTGA	27	20,972–20,998	58.0	2.38	153
cp32-5R	AATTGTCTTGTATAGATTCCAACTTC	26	21,124–21,099	51.5	2.38	
cp32-1F	AAACATTAGTAGAAAGTGAATTTGATTTAC	30	21,109–21,138	51.5	2.38	176
cp32-1R	CCTATGTTCCTTATAAGGCAAGGGC	25	21,284–21,260	57.3	2.38	
cp32-6F	TGGAGATATTAATGGGGTGGCAATT	25	28156–28180	56.5	2.38	197
cp32-6R	CCAACAAGCTATTCCCTTCTACAAT	25	28,352–28,328	55.0	2.38	
cp32-7F	TTCAATGAATCCGGATGATGTTGA	24	18,535–18,558	54.7	2.38	225
cp32-7R	TTATTGTTTAATGCTGTTATATATGC	26	18,759–18,734	48.5	2.38	
cp32-3F	CCATTTATATTCTTAAAGTCGTTTA	25	16,474–16,498	47.5	3.57	251
cp32-3R	TGATCATCACCGCCTTGATCTAAAG	25	16,724–16,700	56.8	3.57	
cp32-12F	TTTGTATCCTTATCTGTATAACCAT	25	16,720–16,744	49.6	2.38	295
cp32-12R	TTGTATCAATGTTATTTGTAATGGC	25	17,014–16,990	50.3	2.38	
cp32-9F	GTATTCTAAGGGACTTAGATAAGT	24	1,341–1,364	49.1	2.38	341
cp32-9R	TGAATACTCTCAGCACTATTGACCT	25	1,681–1,657	55.3	2.38	
cp32-11F	GTTGTTGCCATTATTTGATTTACAG	25	27,718–27,742	51.5	2.38	375
cp32-11R	GAAATTTGTATTGCCTGTGGAGTTA	25	28,092–28,068	53.8	2.38	
cp26F	GGACAATTGGAACGTATCACACAGTA	26	7,094–7,119	56.8	1.58	398
cp26R	TTAAGGCTCTCACAGGAGGCTCCAT	25	7,491–7,467	61.4	1.58	

### Multiplex PCR and gel electrophoresis

PCR was performed by using TaKaRa Ex Taq kit (Clontech, USA). Twenty five microliters of optimized PCR reaction includes 100 ng of boiled whole cell lysate of *B. burgdorferi* (as DNA template), 2.5 μl of 10 × Ex Taq Buffer, 2.5 μl of 10 × dNTP Mixture, 1 μl of Ex Taq DNA polymerase, and 2 μl of 12.5 × primer mix. Given that each primer pair has different amplification efficiency, the concentrations for each primer pair used in the primer mix were adjusted based on the PCR result. For the primer mix to amplify linear plasmids (mixLp), 222 μl of 12.5 × mixlp primer stock includes12 μl of 50 μM primer stock for lp25, lp54, lp28-4, 15 μl for lp38, and 10 μl for lp28-1, lp28-3, lp28-5, lp28-6, lp17, lp36. For the primer mix to amplify circular plasmids (mixCp), 210 μl of 12.5 × mixcp primer stock includes 15 μl of 50 μM primer stock for cp32-3, 10 μl for cp26, 10 μl for cp32-1, cp32-4, cp32-5, cp32-6, cp32-9, cp32-11, cp32-12. The PCR reaction conditions were as the following: (1) 1 cycle of initial denaturation at 94°C for 5 mins; (2) 40 cycles of amplification including denaturation at 94°C for 30 S, annealing at 54°C for 1 min, and extension at 68°C for 1 min; (3) 1 cycle of final extension at 68°C for 5 min. PCR products were separated using 3% metaphor agarose gel (Lonza, Cohasset MN, USA) in TBE buffer (0.089 M Tris base, 0.089 M boric acid, 2 mM EDTA, pH 8.2–8.4) by running 100 V for 1 h and then 80 V for 2 h.

## Results

### Establish a multiplex PCR method for rapid plasmid profiling of *B. burgdorferi* strain 297

Genome sequence revealed that *B. burgdorferi* strain 297 contains at least 10 linear and 10 circular plasmids ranging from 17 to 54 Kb. The published plasmid sequences for strain 297 include 19 plasmids (Schutzer et al., 2011; Casjens et al., 2012). The strain 297 used for sequencing lost linear plasmid, lp25, so lp25 sequence of strain 297 is not available. In *B. burgdorferi* strain B31, lp25 contains at least two genes *pncA* and *bptA*, which are important for the enzootic cycle of *B. burgdorferi* (Purser et al., 2003; Grimm et al., 2004a; Revel et al., 2005). It was reported previously that strain 297 has lp25 (Grimm et al., 2004a; Revel et al., 2005). Of note, two previously reported circular plasmids, cp18-1 and cp18-2 in strain 297 (Caimano et al., 2000), were the result of ~9 Kbp truncation from cp32, and were subsequently renamed as cp32-7 and cp32-9 (Casjens et al., 2012).

**Figure 1:**
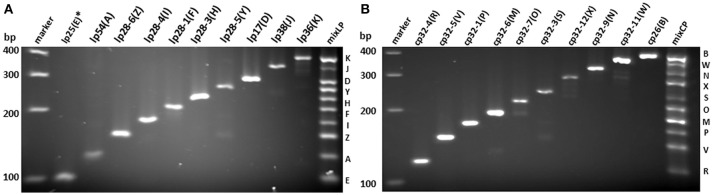
**Verification of PCR primer specificity and efficiency for each endogenous plasmid in *B. burgdorferi* strain 297**. PCR products were separated on 3% metaphor agarose gel. Each product for linear **(A)** and circular **(B)** plasmids with corresponding designation were labeled on top. The mixed product from pooled primer mix for linear plasmids (mixLP) and circular plasmids (mixCP) were on the right of each gel, and the band corresponding to each plasmid is labeled with designated letter on the right. DNA ladder is labeled on the left. ^*^The primer sequences for lp25(E) were based on *bptA* sequence.

All PCR primer pairs specific for each endogenous plasmid were designed based on multi-alignment of all published plasmid sequences of *B. burgdorferi* strain 297 (Schutzer et al., 2011), followed by BLAST analyses against published *B. burgdorferi* B31 chromosomal sequences (see Materials and Methods). Since the sequence of lp25 of strain 297 is not available, the primer pair for lp25 of strain 297 was designed based on the *bptA* gene sequence from lp25 of strain B31 (Revel et al., 2005). The specificity and efficiency of each primer pair for corresponding plasmid were confirmed by regular PCR (Figures 1A,B). Then, primer pairs for all linear plasmids or circular plasmids were mixed together (mixLp or mixCp), and two multiplex PCR reactions were performed accordingly. The sizes of PCR products in each of the two groups were designed to differ at least 20 bps, so that they could be readily separated by gel electrophoresis (Figure 1).

### Comparison of plasmid contents among *B. burgdorferi* clones with different OspC levels

**Figure 2:**
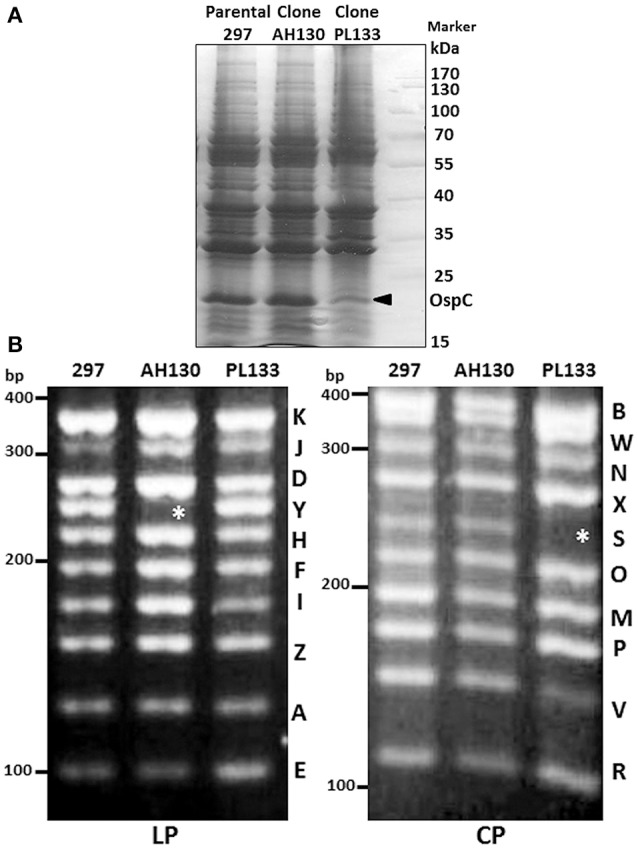
**Protein profile and plasmid content of *B. burgdorferi* strain 297 clones AH130 and PL133. (A)** SDS-PAGE gel. *B. burgdorferi* strain 297 clones AH130 and PL133 were cultivated at 37°C and harvested at stationary phase of growth. The band corresponding to OspC is indicated by arrow. **(B)** Plasmid content of linear plasmid (LP) and circular plasmid (CP). Asterisk (^*^) indicates absent plasmids.

Having established a rapid plasmid profiling method specific for strain 297, we sought to examine the plasmid contents of some commonly used clones of *B. burgdorferi* strain 297. Clone AH130 was isolated on semi-solid agar plate from the parental low passage *B. burgdorferi* strain 297 (Yang et al., 2004). Clone PL133 was isolated from mice after repeated rounds of needle inoculation by culturing ear-punch biopsies (Revel et al., 2005; Blevins et al., 2007). Although both AH130 and PL133 are infectious in mice, they produced different levels of OspC under the *in vitro* cultivation conditions: AH130 produces very high level of OspC at 35 or 37°C at late logarithmic phase of growth (Yang et al., 2004; Gilbert et al., 2007; Ye et al., 2016a), even without performing the temperature shift from 23 to 35°C, a condition often required for OspC induction in strain B31 (Schwan and Piesman, 2000). On the other hand, PL133 had relative low level of OspC (Figure 2A).

Given that certain gene(s) on endogenous plasmids can affect OspC levels of *B. burgdorferi* (Sarkar et al., 2011), it is possible that AH130 and PL133 may have different plasmid content when isolated from parental 297 strain, resulting in a phenotype of different OspC expression. Although AH130 and PL133 were thought to have a complete plasmid content, the previous work was done using B31 plasmid sequences since the 297 plasmid sequences was not available till recently. Upon examining the plasmid content, we found that AH130 and PL133 actually do not have a complete plasmid content: AH130 lost the linear plasmid lp28-5, whereas PL133 lost a circular plasmid cp32-3 (Figure 2B). These results suggest that (1) the parental *B. burgdorferi* strain 297 is a mixed clone; (2) the difference in plasmid contents may contribute to the difference in OspC production between AH130 and PL133.

### Absence of lp28-5 is not associated with high OspC level

**Figure 3:**
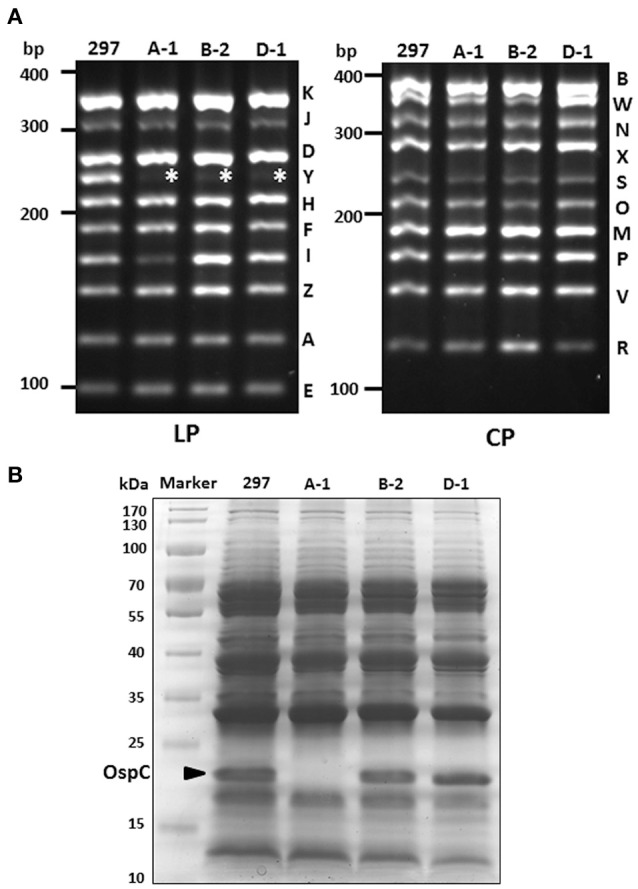
**Plasmid profiles of additional clones with high or low OspC levels. (A)** Plasmid profile. Clones A-1, B-2, D-1 lost lp28-5 (indicated by asterisk). LP, linear plasmid; CP, circular plasmid. **(B)** SDS-PAGE. Spirochetes were cultivated at 37°C and harvested at stationary phase of growth. The band corresponding to OspC is indicated by arrow.

AH130 expressed very high level of OspC. One possibility is that lp28-5 may encodes a gene that is inhibitory to *ospC* expression, and absence of lp28-5 leads to high OspC level. Given that the parental clone 297 contains mixed clones with OspC high or low phenotypes, one cannot draw conclusion from parental 297 that the presence of lp28-5 can also have high OspC level. Thus, we investigated whether absence of lp28-5 leads to high OspC level by screening 297 clones isolated from semi-solid agar plate that lack lp28-5, and identified three such clones (A-1, B-2, D-1) (Figure 3A). While two lp28-5-lacking clones (B-2, D-1) exhibited high OspC level, one clone (A-1) had no detectable level of OspC on the Coomassie stained SDS gel (Figure 3B). This data suggests that absence of lp28-5 does not cause high level of *ospC* expression. Regarding the potential association between loss of cp32-3 and the low OspC level observed in PL133, we screened more than 50 clones to identify additional 297 clone lacking cp32-3 but were not successful.

### Different levels of RpoS and BosR among *B. burgdorferi* 297 clones

**Figure 4:**
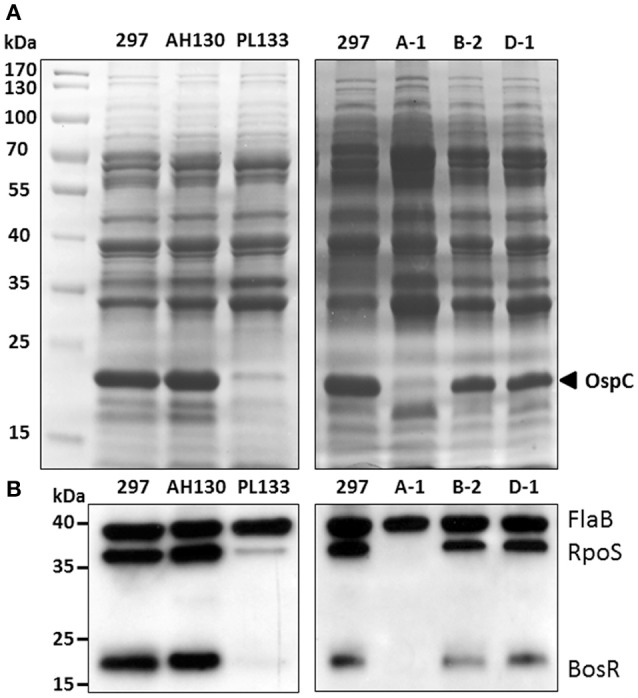
**Levels of RpoS and BosR in clones with different OspC levels. (A)** SDS-PAGE. The band corresponding to OspC is labeled at the right. **(B)** Immunoblot. Whole cell lysates of *B. burgdorferi* clones were separated and probed with a mixture of α-FlaB, α-RpoS and α-BosR monoclonal antibodies. FlaB serves as an internal control.

To further investigate what may contribute to the *ospC* expression variation among strain 297 clones, we examined the upstream regulators that govern *ospC* expression. *OspC* has a RpoS-type promoter which is controlled by RpoS (Hübner et al., 2001; Yang et al., 2005). As shown in Figures 4A,B, the low OspC clones, PL133 and A-1, had very low or undetectable levels of RpoS in comparison to high OspC clones (AH130, B-2, D-1). This result indicates that variation in OspC levels is due to the difference in RpoS levels in *B. burgdorferi* 297 clones.

Many factors can influence *rpoS* and *ospC* expression such as Rrp2, RpoN, BosR, BadR, Rrp1, DsrA, BtmA, SodA, and BBD18 (Lybecker and Samuels, 2007; Hyde et al., 2009; Ouyang et al., 2009, 2011; Rogers et al., 2009; Miller et al., 2013; Sze et al., 2013; Troxell et al., 2013; Dulebohn et al., 2014; He et al., 2014; Esteve-Gassent et al., 2015; Ouyang and Zhou, 2015). In particular, expression of *rpoS* is directly activated by the alternative factor RpoN as well as the transcriptional factor, BosR. Activation of the RpoN pathway is governed by phosphorylation of the enhancer binding protein Rrp2 (Yang et al., 2003; Boardman et al., 2008). We recently showed that Rrp2 and RpoN is likely constitutively active under *in vitro* cultivation conditions, since phosphorylation of Rrp2 is vital for *B. burgdorferi* replication and Rrp2 is constitutively phosphorylated during *in vitro* replication (Groshong et al., 2012; Yin et al., 2016). Thus, Rrp2 is unlikely the factor that contributes to *ospC* expression variation among *B. burgdorferi* strains. Thus, we focused on the second factor, BosR. As shown in Figure 4B, PL133 and A-1 had very low or undetectable levels of BosR in comparison to high OspC clones (AH130, B-2, D-1). These results suggest that there is a difference in BosR levels among *B. burgdorferi* strains, which results in different levels of RpoS, and subsequently, *ospC* expression variation.

## Discussion

*B. burgdorferi* strain 297 is one of the widely used strain in borrelial research, especially for studying differential gene expression. Lack of a plasmid profiling method have made it difficult to track the plasmid contents of strain 297 during *in vitro* growth and genetic manipulation. Herein, we developed a multiplex PCR method that allows rapid plasmid profiling of strain 297. With such method, we found that the parental 297 strain is likely a mixed strain. Two of the commonly used infectious clones of strain 297, AH130 and PL133, which were previously thought having a complete endogenous plasmid content based on previous plasmid analysis using B31 plasmid sequences-derived primers, actually lost lp28-5 and cp32-3, respectively. We further showed that the difference in plasmid profiles among *B. burgdorferi* clones could not explain *ospC* expression variation among the clones. On the other hand, we observed that there is a difference in BosR levels among the clones, which contributes to *ospC* expression variation.

Tracking each endogenous plasmid during every step of genetic manipulation of *B. burgdorferi* by PCR is time-consuming. Previously, Bunikis et al., developed a multiplex PCR as a tool for validating plasmid profiling of *B. burgdorferi* strain B31, which has significantly improved the efficiency of the procedure of plasmid profiling (Bunikis et al., 2011). Unfortunately, the optimized primer pairs for plasmid profiling of strain B31 cannot be applied for other *B. burgdorferi* strains such as strain 297, due to sequence variation of endogenous plasmids among *B. burgdorferi* strains. The rapid plasmid profiling specifically for strain 297 reported in this study should be widely useful for borrelial research whenever *B. burgdorferi* 297 is used.

The data presented here shows that lp28-5 does not contribute to *ospC* expression variation among *B. burgdorferi* strains. Although we were unable to identify other clones missing cp32-3, the redundancy of cp32 plasmids in *B. burgdorferi* makes it unlikely that loss of cp32-3 is responsible for the variation in OspC expression. Thus, this study suggests that other yet-to-be identified genetic differences between the two strains contributes to OspC expression variation. On the other hand, this result does not conclude that endogenous plasmids do not play a role in regulation of *ospC* expression. In fact, *bbd18* on lp17 of *B. burgdorferi* strain B31 plays a negative role in regulation of *rpoS* and *ospC* expression (Sarkar et al., 2011; Dulebohn et al., 2014). The 297 strain also contains a similar lp17 plasmid. lp17 is one of the endogenous plasmids that are well-retained by *B. burgdorferi* under *in vitro* cultivation (Purser and Norris, 2000), so it is not surprising that we did not find a 297 clone lacking this plasmid.

The pattern of protein expression in the low OspC expressing clone A1 varied in different preparations and was sometimes significantly different from that seen in the high OspC expressing clones. For example, we noticed an increased intensity of a band below OspC in clone A1 in Figure 4A, but not in Figure 3. It has previously been proposed that downregulation of OspC is accompanied by upregulation of other lipoproteins (He et al., 2008; Xu et al., 2008). It is noteworthy that different clones derived from isolates of *B. burgdorferi* strain B31 have been shown to have considerable heterogeneity not only in protein profile and plasmid content, but also in colony phenotype on solid agar (Elias et al., 2002). We screened and compared strain 297 clones with different colony morphologies, and found that colony morphology is not associated with plasmid content (data not shown).

In addition, *B. burgdorferi* strain B31 presents different types of colony morphology on the semi-solid agar plate. Thus, we also screened and compared groups with different colony morphologies, and found that colony morphology is not associated with plasmid content (data not shown).

BosR is a FurR/PerR homolog (for review, see, Samuels and Radolf, 2009). It is required for activation of *rpoS* expression in *B. burgdorferi*, but the mechanism remains unknown. Recently, it was reported that BosR undergoes autoregulation (Ouyang et al., 2016). However, regulation of *bosR* expression in response to environmental signals by large remains unknown. The study herein demonstrates that the BosR levels are highly variable among *B. burgdorferi* strains. Continuing efforts on the investigation of clonal differences will likely help uncover the mechanism of regulation of BosR by environmental factors.

## Ethics statement

The work conducted in the manuscript was approved by Indiana University School of Medicine Biosafety Committee (protocol number 643).

## Author contributions

XX, YY, XY, YL conceived and designed the experiments; XX, JD, and TL performed the experiments; XX and TC analyzed the data; XX, YL, and XY wrote the paper. XX and YY contributed equally to this work.

### Conflict of interest statement

The authors declare that the research was conducted in the absence of any commercial or financial relationships that could be construed as a potential conflict of interest.
